# 260. Corticosteroid treatment in influenza virus and SARS-CoV-2 co-infected patients

**DOI:** 10.1093/ofid/ofac492.338

**Published:** 2022-12-15

**Authors:** Maaike C Swets, Clark Russell, Ewen Harrison, Annemarie Docherty, Nazir Lone, Michelle Girvan, Hayley Hardwick, Leonardus Visser, Peter Openshaw, Geert Groeneveld, Calum Semple, Kenneth Baillie

**Affiliations:** Leiden University Medical Center, Amsterdam, Noord-Holland, Netherlands; University of Edinburgh, Edinburgh, Scotland, United Kingdom; University of Edinburgh, Edinburgh, Scotland, United Kingdom; University of Edinburgh, Edinburgh, Scotland, United Kingdom; University of Edinburgh, Edinburgh, Scotland, United Kingdom; University of Liverpool, Liverpool, England, United Kingdom; University of Liverpool, Liverpool, England, United Kingdom; Leiden University Medical Centre, Leiden, Zuid-Holland, Netherlands; Imperial College London, London, England, United Kingdom; Leiden University Medical, Leiden, Zuid-Holland, Netherlands; University of Liverpool, Liverpool, England, United Kingdom; University of Edinburgh, Edinburgh, Scotland, United Kingdom

## Abstract

**Background:**

Co-infections with SARS-CoV-2 and influenza virus may become more prevalent now that many countries are easing restrictions to reduce the spread of SARS-CoV-2. Co-infected patients are more likely to receive invasive mechanical ventilation (IMV) and have higher odds of in-hospital mortality. In the RECOVERY trial, dexamethasone was found to reduce the risk of 28-day mortality in hospitalised COVID-19 patients. On June 16, 2020, corticosteroids were included in clinical guidelines for the treatment of COVID-19 patients requiring supplemental oxygen. However, corticosteroid treatment in severe influenza virus infection may increase mortality. The effect of steroids in influenza and COVID-19 co-infected patients is unknown.

**Methods:**

Adult patients with RT-PCR confirmed SARS-CoV-2 and influenza virus co-infection were evaluated. Patients without supplemental oxygen during admission were excluded. Patients who were hospitalised prior to June 16, 2020 were included in the ‘early’ group and patients who were hospitalised on or after June 16, 2020 were included in the ‘late’ group.

**Results:**

171 co-infected patients were included, 123 patients in the early group (table 1) and 48 in the late group (table 2).

In the early group, 25 patients received steroids. In the late group, 40 patients received steroids.

In the early group, the proportion of patients who were admitted to critical care was slightly lower in the group that received steroids. IMV was similar in both groups. In-hospital mortality was slightly higher in the group treated with steroids.

In the late group, critical care admission and receipt of IMV were higher in the group not treated with corticosteroids than the group with corticosteroid treatment. In-hospital mortality was slightly lower in the group not treated with steroids.

**Figure:**
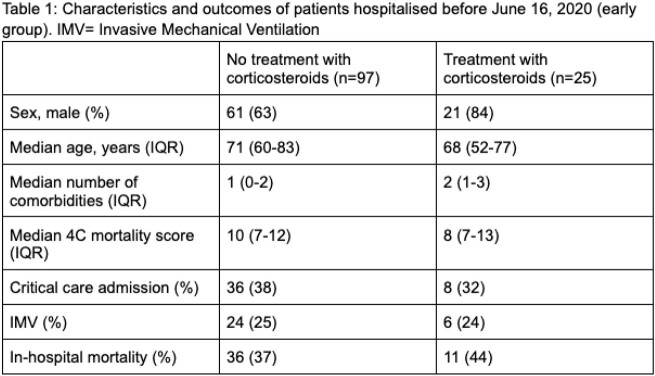

**Figure:**
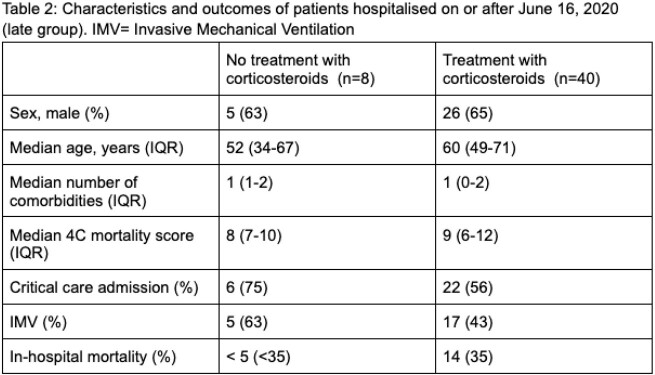

**Conclusion:**

There are differences between co-infected patients who were treated and not treated with corticosteroids and differences between the early and late groups.

A limitation is that no dates were collected for the start of steroid treatment, making it impossible to draw conclusions on the causality of the need for IMV and treatment with steroids in this analysis. Future research should focus on the effect of steroids in COVID-19 and influenza co-infected patients.

**Disclosures:**

**Peter Openshaw, PhD**, Bavarian Nordic: Advisor/Consultant|Cepheid: Advisor/Consultant|GlaxoSmithKline: Advisor/Consultant|Janssen: Advisor/Consultant|Pfizer: Advisor/Consultant **Calum Semple, PhD**, Integrum Scientific: Scientific Advisory Board|Integrum Scientific: Stocks/Bonds.

